# Spectral Power Distribution of Heart Rate Variability in Contiguous Short-Term Intervals

**DOI:** 10.7759/cureus.67221

**Published:** 2024-08-19

**Authors:** Harvey N Mayrovitz

**Affiliations:** 1 Medical Education, Nova Southeastern University Dr. Kiran C. Patel College of Allopathic Medicine, Davie, USA

**Keywords:** sequential changes, temporal changes, analysis, total power, minimum detectible change, spectral power, hrv, heart rate variability

## Abstract

Introduction: Heart rate variability (HRV) is determined by the variation of consecutive cardiac electrical excitations, usually from RR intervals of an EKG. The sequence of intervals is a time series that yields three HRV parameter categories: time domain, frequency domain, and nonlinear. Parameter estimates are based on widely different EKG sample times: short-term (~5-10 minutes), longer (24 hours), and ultra-short (<5 minutes). Five-minute intervals are useful to evaluate intervention effects that change HRV in a single session by comparing pre-to-post values. This approach relies on knowing the minimal detectible change (MDC) that indicates a real change in clinical and research studies. The specific aims of this pilot study were to (1) evaluate HRV power and its spectral distribution among contiguous five-minute intervals, (2) compare the power distribution in a five-minute interval with a full 45-minute assessment, and (3) provide data to aid estimation of the MDC between pre- and post-interventions during a single session.

Methods: Twelve self-reported healthy young adults participated after signing an approved consent. Participation required subjects who had no history of cardiovascular disease or were taking vasoactive substances. Persons with diabetes were not eligible. While subjects were supine, EKG leads were placed, and EKG was recorded for 45 minutes at 1000 samples/sec. The 45 minutes were divided into nine five-minute contiguous intervals, and the spectral density in each was determined. Total power and spectral percentages within each interval were assessed in the very low (VLF, 0.003-0.04 Hz), low (LF, 0.04-0.15 Hz), and high (HF, 0.15-0.4 Hz) frequency bands. These were compared among intervals and to the full 45-minute sample. The MDC was determined by comparing powers in five-minute intervals separated by 10 minutes. The standard error of the measurement (SEME) for each pair was calculated from the square root of the mean square error (√MSE). MSE was based on a two-factor analysis of variance, and MDC was 2×√2×SEME.

Results: Differences in total power and spectral power distribution among intervals were not statistically significant. The total mean power±SD was 4561±1434 ms². The maximum difference in total power was 7.85%. The mean power for the VLF, LF, and HF bands was respectively 1713±1736 ms², 1574±1072 ms², and 1257±1016 ms². The maximum percentage difference in spectral power across all intervals for VLF, LF, and HF was respectively 3.75%, 8.5%, and 7.4%. The percentage of power in the VLF, LF, and HF bands was respectively 37.9%, 36.1%, and 25.9%. The ratios of spectral to total power for VLF, LF, and HF bands were respectively 0.80±0.07, 1.20±0.11, and 1.22±0.10. MDC percentage values were 21.0±4.9% for the HF band, 25.7±1.4% for the LF band, and 30.4±5.5% for the VLF band.

Conclusion: Results offer initial estimates of variations in HRV power in the VLF, LF, and HF bands in contiguous five-minute intervals and estimates of the minimum detectible “real” changes between intervals separated by 10 minutes. The pattern of variation and data are useful in experimental planning in which HRV spectral power changes are assessed subsequent to a short-duration intervention during a single session. MDC values (21.0% in the HF band to 30.4% in the VLF band) provide initial estimates useful for estimating the number of participants needed to evaluate the impact of an intervention on spectral components of HRV.

## Introduction

Heart rate variability (HRV) is a measure of the temporal variation between consecutive cardiac electrical excitations, usually determined by measurements of consecutive RR intervals from an EKG. The sequence of these RR intervals, usually measured in seconds, represents a time series in which RR times are presented as a function of time and can be analyzed using methods suitable for time series analysis. The preponderance of analysis types permits the assessment of three broad categories of HRV parameters. One is expressed as time domain parameters, such as the standard deviation of the time difference between consecutive RR intervals [1-3]. Another is expressed as frequency domain parameters, which are based on transforming aspects of the time series into the frequency domain, leading to parameters that include spectral power within different frequency ranges [4-6]. A third approach analyzes the process without assuming linearity, as is done with the first two approaches, and applies nonlinear analyses to yield parameters based on these analyses [7-9]. Current approaches to assess these HRV parameters are based on time samples of widely different extents. Acquired data durations from which HRV parameters are determined to include short-term, about 5-10 minutes [10-12], longer-term, including 24 hours, and even ultra-short-term, with sample times less than five minutes or less than a minute [13-15].

The use of HRV to assess multiple aspects of physiological functions and their changes with age and disease states has skyrocketed in recent years. Evidence for this can be found in the increase in the number of peer-reviewed published papers containing the term “heart rate variability” in the title. A Web of Science database search, done recently by the author (July 15, 2024), covering the years from 1983 to 2023, indicated only 17 papers in 1983 compared to 663 in 2023, a 39-fold increase. To account for a generalized increase in publishing over these years, a comparison was made with papers published with the term “blood pressure” in the title. It was found that there were 570 articles in 1983 and 2,462 in 2023, a 4.3-fold increase, which is nine times less than for HRV articles. This increase in the use of HRV parameters in both clinical and research applications emphasizes the importance of expanding our understanding of HRV parameter variations in relation to interpreting pre-to-post-intervention HRV changes.

Over this time frame, the clinical applications of HRV and the methods used to quantitatively assess it have evolved and expanded [16,17]. Short-term assessments, of about five minutes, are often used to evaluate intervention effects based on changes in HRV parameters in a single session by comparing pre-to-post-intervention HRV parameters [18-20]. However, determining if HRV changes after an intervention during a single session are due to the intervention depends in part on knowledge of the expected normal temporal variability in the HRV parameter being evaluated. This, in turn, depends on the empirical temporal variation of HRV parameters that occur within contiguous five-minute intervals, separated by the time between a baseline pre-intervention and post-intervention. Systematic characterization addressing this specific issue is lacking in the literature. As a consequence, there is insufficient information available to allow for a data-based estimate of the minimum amount of change that can properly be judged as an intervention effect during a single session. The broad focus of the present pilot study was to characterize the temporal sequential changes in the short-term spectral power of HRV during a single session. The specific aims were to (1) gain insight into the variation of total HRV power and its spectral distribution among contiguous five-minute intervals, (2) evaluate the difference in power distribution in a given five-minute interval from that of a full 45-minute assessment during a single session, and (3) provide data to aid in the estimation of the minimum detectible change (MDC) between pre- and post-interventions during a single session.

## Materials and methods

Subjects

A convenience sample of 12 adult subjects (six male), recruited from first-year medical students, participated in this pilot research study after each participant signed an Institutional Review Board-approved informed consent #2019-598-NSU. To be eligible for participation in this research study, participants needed to indicate that they were free of any known neurological or cardiovascular condition, were not taking any medication that might affect heart rate and were willing and able to lie supine for one hour. Persons with diabetes were not eligible to participate. For the participating group, age (mean±SD) was 25.9±1.4 years; systolic and diastolic blood pressure were 120.8±7.7 mmHg and 73.5±5.5 mmHg, respectively; and body mass index (BMI) was 23.2±2.8 kg/m². 

Procedures

When subjects arrived at the laboratory, they lay supine on a padded examination table with their head supported by a pillow. All procedures were scheduled at normal lunch times, between 1200 and 1330 hours. While the subject was resting, electrodes for a single-lead EKG measurement (lead I) were placed on each leg, on the lateral aspect, approximately 5 cm proximal to the lateral malleolus. In addition, one electrode was placed on the dorsum of the left wrist. The electrodes were connected to an EKG amplifier (EGC100C; Biopac Systems Inc., Goleta, CA, USA). The amplifier was set to normal mode, with the low-pass filter set to 35 Hz, the high-pass filter set to 1 Hz, and the gain set to 1000. The signal output of the amplifier was coupled to a data acquisition system device (DL-710-UL; DataQ Instruments, Akron, OH, USA) and was sampled at 1000 samples/sec. After lying supine for 10 minutes, the recording of the EKG signal began and continued for 45 minutes continuously. During this time, the room lights were dimmed, talking was prohibited, and other disturbances were minimized. At the end of the recording interval, after the participant had been lying for about 55 minutes, the subject's blood pressure was measured in their left arm with an automatic blood pressure system (HEM-711; Omron Healthcare, Sunrise, FL, USA).

HRV analysis

**Figure 1:**
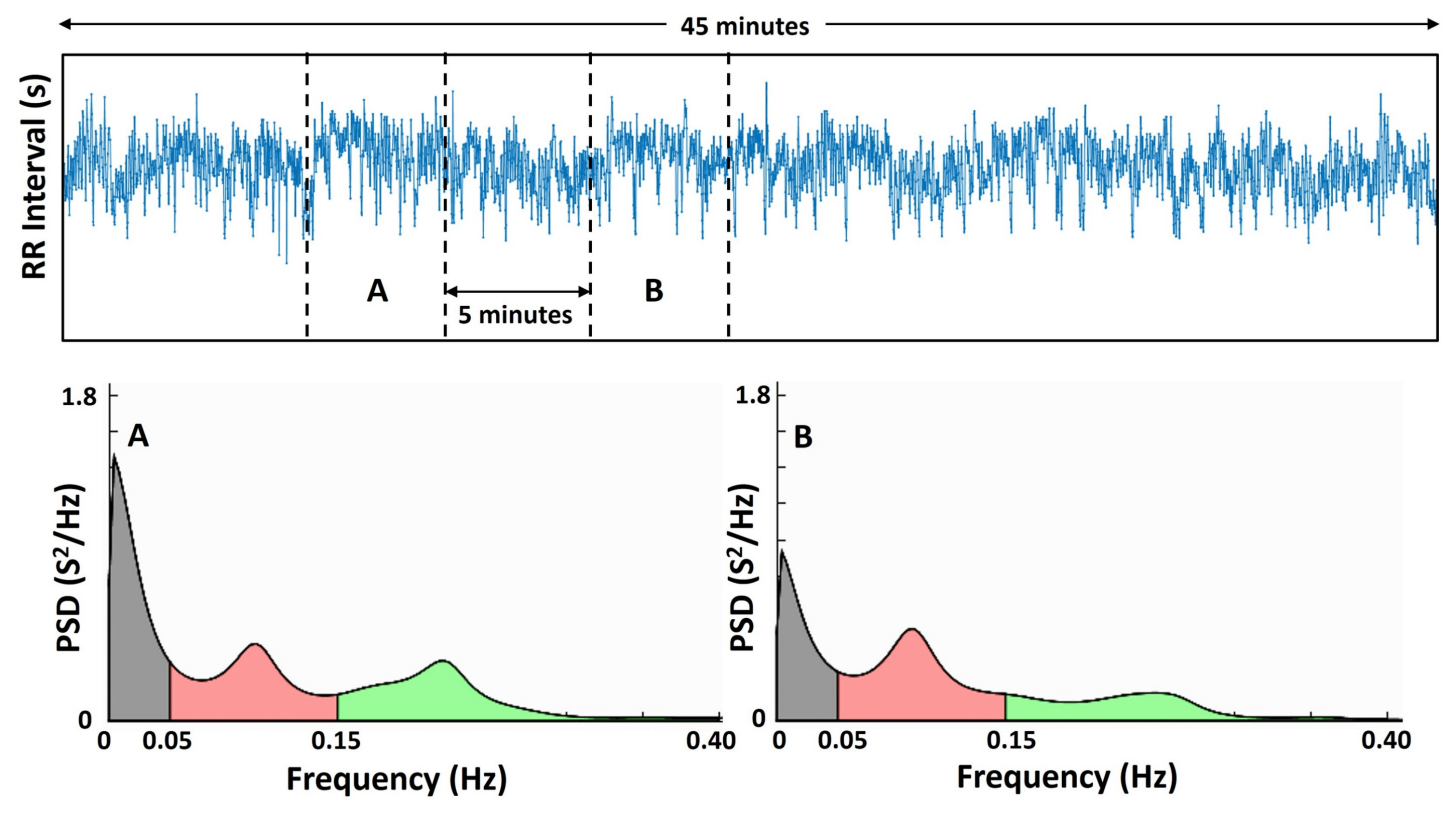
Determination of power spectral distributions The top panel shows a 45-minute time series of consecutive RR intervals derived from a subject’s electrocardiogram (EKG). The vertical dashed lines indicate five-minute segments. The bottom panel shows the calculated power spectral density (PSD) corresponding to sections A and B in the upper panel. The three frequency bands of interest are the very low frequency (VLF), from 0.003 to 0.05 Hz; the low-frequency band (LF), from 0.05 to 0.15 Hz; and the high-frequency band (HF), from 0.15 to 0.40 Hz. The nine individual five-minute intervals are analyzed separately for each subject.

The 45-minute recording was divided into nine five-minute contiguous intervals, constituting nine short-term sequential HRV intervals. Before performing spectral analysis, each 45-minute recording was manually reviewed for the presence of ectopic impulses or other EKG irregularities. For this young, healthy group, none were found, and thus no corrections for such ectopic signals were required. The consecutive RR interval durations were determined by peak detecting the R-waves and then calculating the duration between peaks using software (Advanced CODAS Analysis Software; DataQ Instruments, Akron, OH, USA). The adequacy of the automated peak detection process was inspected visually, and any missing or added detections were manually corrected. The time series of consecutive RR intervals were then processed for each of the nine contiguous five-minute segments using dedicated software for HRV analysis (Kubios HRV standard v3.1.0) [21]. The Kubios software was chosen because of its long history of wide use for HRV analysis and the fact that it is freely available. To illustrate, one such time series is shown in Figure 1. In the figure, spectral power distribution in each of the three standardized frequency bands is shown. These bands are the very low-frequency band (VLF, 0.003-0.04 Hz), the low-frequency band (LF, 0.04-0.15 Hz), and the high-frequency band (HF, 0.15-0.4 Hz) [21]. Power is expressed in units of ms², and when used, the power spectral density (PSD) is in units of ms²/Hz.

Variability analysis

Total and spectral power among intervals were tested for normality using the Shapiro-Wilk test. Each power parameter was tested for each of the nine intervals. For total power, p-values ranged from <0.001 to 0.354, with five intervals having p-values <0.05. For VLF power, p-values ranged from <0.001 to 0.276, with seven intervals having p-values <0.05. For LF power, p-values ranged from 0.014 to 0.436, with four intervals having p-values <0.05. For HF power, p-values ranged from 0.011 to 0.168, with four intervals having p-values <0.05. Based on the test results, normality could not be assumed, so differences in total power and spectral power among the nine five-minute intervals were tested for statistical significance using the nonparametric Friedman test. The Friedman test is an appropriate nonparametric alternative to one-way repeated measures analysis of variance, needed since the data are not normally distributed. Paired differences in power between spectral bands (VLF, LF, and HF) within each interval were tested for statistical significance using the nonparametric Wilcoxon test, with a p-value of <0.01 used as the threshold for significance. In addition, to estimate the maximum variability among intervals, the maximum percentage difference among intervals was calculated for total power and for each spectral band.

Estimate of MDC

The premise of this estimate is based on a five-minute sample taken prior to a hypothetical intervention that lasts for 10 minutes, and an immediate post-intervention five-minute sample. The reason for choosing a separation of 10 minutes was twofold. Firstly, it allowed for the subsequent use of six pre-post assessments of the assumed 10-minute intervention, as described subsequently. Secondly, it was viewed that 10 minutes would be sufficient for some types of short-term interventions to be implemented, for which the effect on HRV was of interest. To utilize the obtained data set of nine contiguous five-minute intervals for this analysis, six pairs of hypothetical pre- and post-intervention intervals were used: intervals 1 and 4, 2 and 5, 3 and 6, 4 and 7, 5 and 8, and 6 and 9. In this way, pre- and post-sampling intervals were each separated by 10 minutes. For each pair, the standard error of the measurement (SEME) was calculated from the square root of the mean square error (√MSE), determined from a two-factor analysis of variance [22]. The MDC was estimated as 2×√2×SEME [23]. This analysis was done separately for each frequency band, with each band’s power (PVLF, PLF, and PHF) relative to the total power in the interval calculated and expressed as a percentage. This percentage was used as the assessment parameter of interest for the hypothetical intervention.

## Results

Distribution of total power among sequential intervals

**Figure 2:**
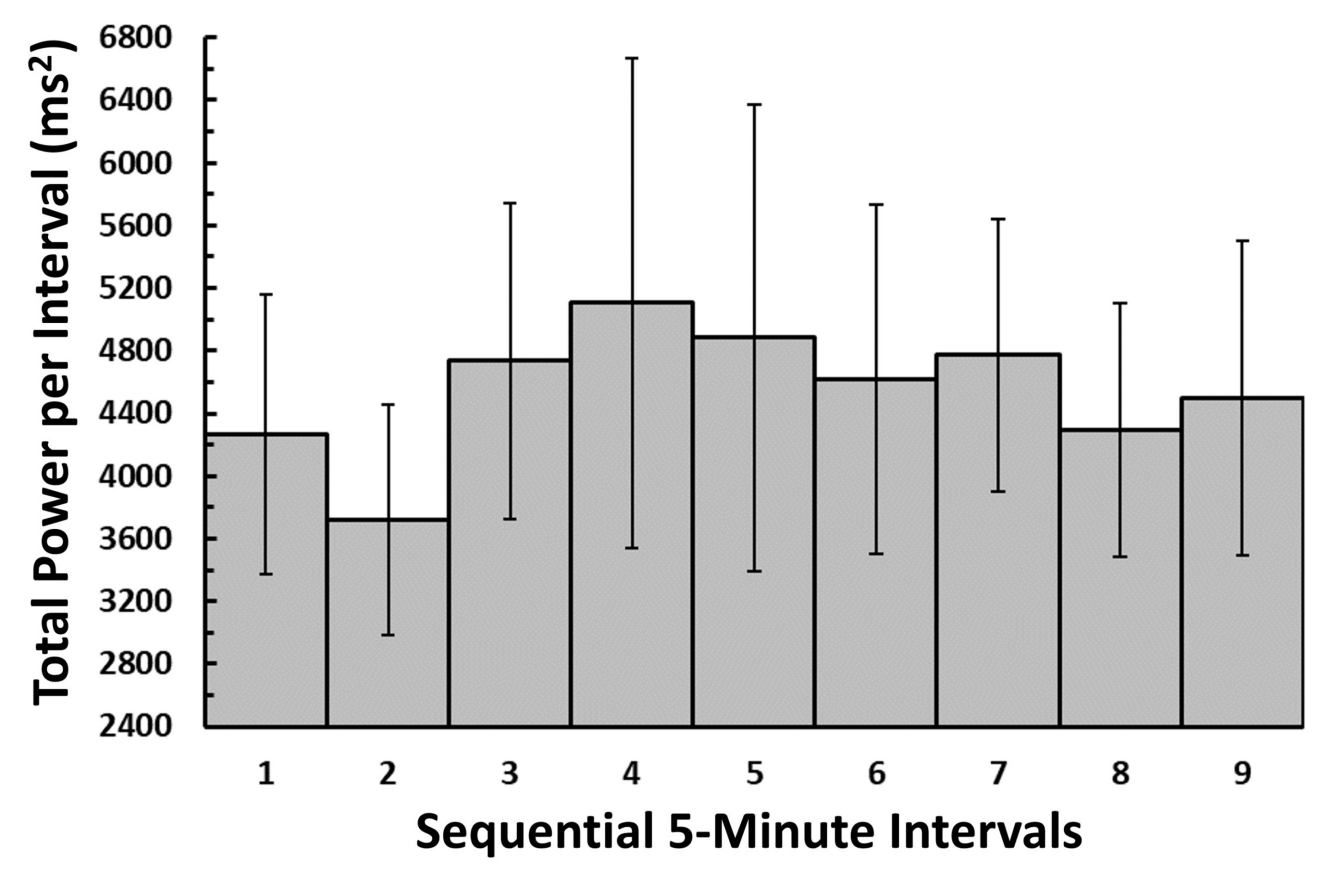
Distribution of absolute total power among sequential intervals The bar heights indicate the total power in each of the contiguous intervals, which consists of the sum of the powers in the very low-frequency band (VLF), low-frequency band (LF), and high-frequency band (HF). The error bars represent the standard error of the mean (SEM). Differences in total power among intervals are not statistically significant, based on the Friedman nonparametric test (p=0.385).

Figure 2 depicts the overall distribution of total power among the nine sequential five-minute intervals. Differences in total power among intervals were not statistically significant, based on the Friedman nonparametric test (p=0.385). Considering all intervals, the overall mean power±SD was 4561±1434 ms². The maximum percentage difference in means is 7.85%, which occurs between segment 2 and segment 4. The absence of a statistical difference among the intervals in total power, combined with the low maximum percentage difference, is encouraging from the perspective of possibly using this parameter as a suitable test parameter in comparing potential intervention changes.

Distribution of power among frequency bands

**Figure 3:**
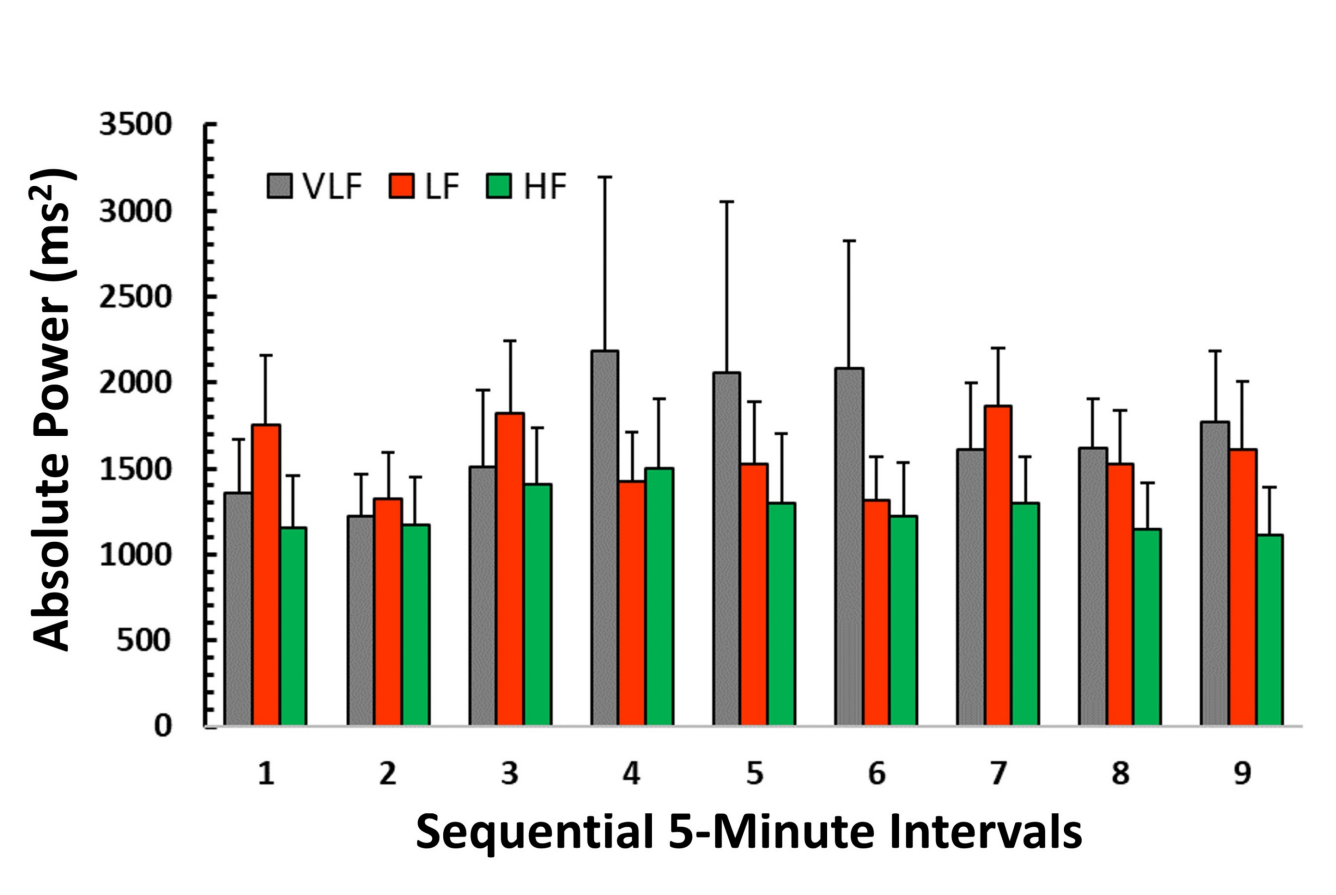
Distribution of absolute power among frequency bands The bar heights indicate the power in each of the three frequency bands for each sequential interval. The error bars represent the standard error of the mean (SEM). In most intervals, the high-frequency power (HF) tends to be lower than either the very low-frequency power (VLF) or the low-frequency power (LF), but differences in power among bands are not statistically significant.

Figure 3 depicts the spectral distribution of power within each of the nine sequential five-minute intervals. The power within each spectral band did not statistically differ among the nine intervals based on Friedman tests for the VLF band (p=0.358) or the LF band (p=0.081) but was marginally significant for the HF band (p=0.041). The marginal significance of the HF band may be partly attributable to its lower mean value in all but one interval (interval 4). However, no observable trend in HF power, or in the other frequency bands, over the nine intervals is apparent. Considering all intervals, the overall mean power±SD for the VLF, LF, and HF bands was 1713±1736 ms², 1574±1072 ms², and 1257±1016 ms², respectively. The maximum percentage difference in mean spectral power across all intervals for VLF, LF, and HF was 3.75%, 8.5%, and 7.4%, respectively.

Spectral distribution of power percentages across intervals

**Figure 4:**
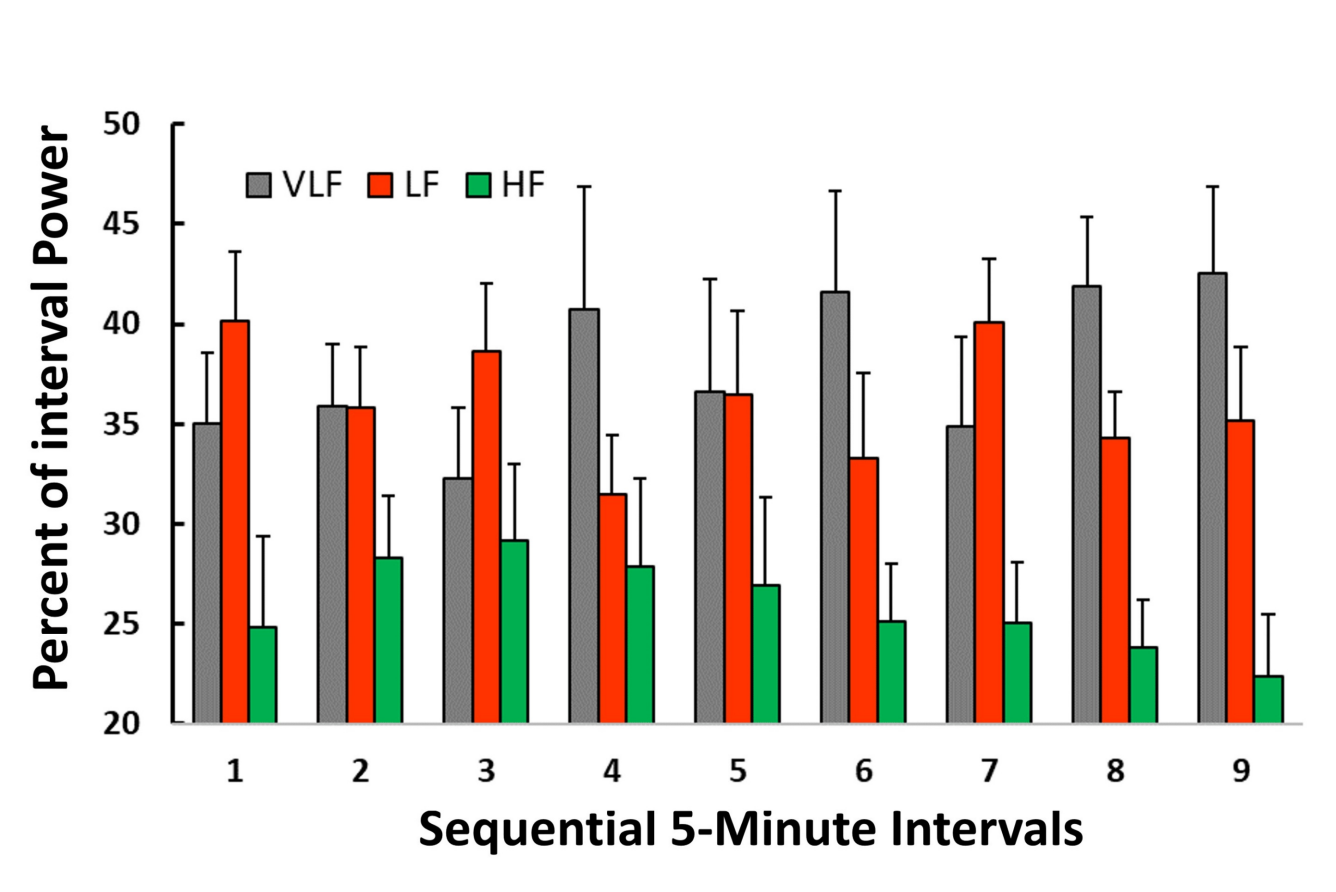
Percentage of power within intervals The bar heights indicate the percentage of interval power in each of the three frequency bands. For any interval, the sum of the band powers equals 100%. The error bars are the standard error of the mean (SEM). In all intervals, the percentage of power within the high-frequency band (HF) tends to be lower than that of either the very low-frequency band (VLF) or the low-frequency band (LF).

Figure 4 depicts the percentage of power attributable to the three frequency bands within each interval. In all intervals, the percentage of power within the HF band tends to be lower than that of either the VLF or LF bands. However, for each frequency band, the percentage of power within each interval did not statistically differ among the nine intervals. Considering all nine intervals, the average percentage of power in the VLF, LF, and HF bands was 37.9%, 36.1%, and 25.9%, respectively. Examination of Figure 4 indicates an apparent increase in HF power percentage during the first three intervals, followed by a progressive decline in the subsequent six intervals. However, no particular significance can be attached to this trend, as there is an overlap of SEMs between adjacent intervals. No specific pattern is observable in either the VLF or LF bands.

Comparison of 5- and 45-minute sequence intervals

**Figure 5:**
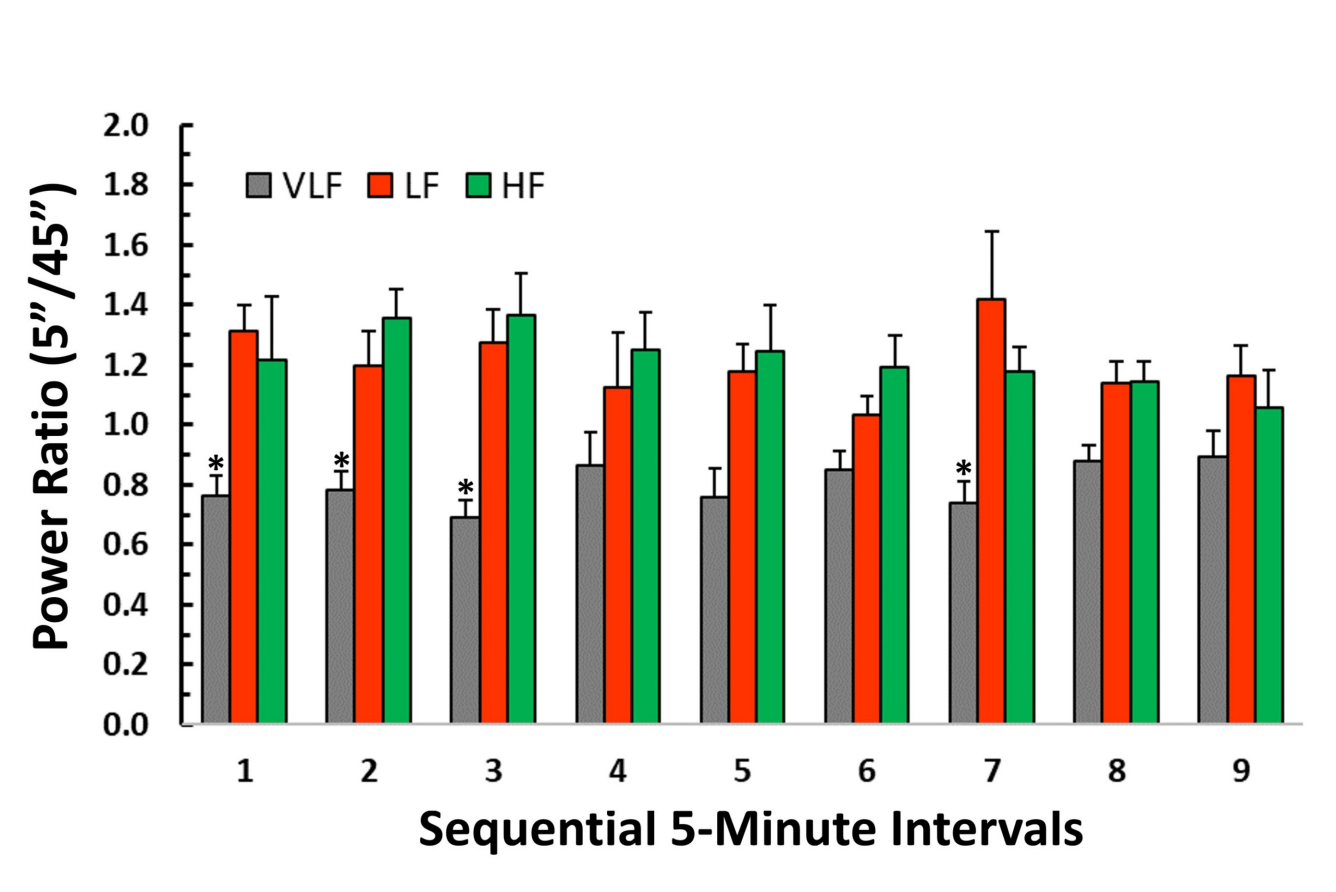
Ratio of five-minute interval power to full 45-minute power The bar heights indicate the ratio of the spectral power distribution in an interval to that of the full 45-minute sampling interval. The error bars represent the standard error of the mean (SEM). In all intervals, the very low-frequency band ratio (VLF) tends to be lower than those of either the low-frequency (LF) or high-frequency (HF) bands. * Indicates that the VLF ratio is statistically lower than either the LF or HF bands (p<0.01).

Figure 5 depicts the interval spectral power normalized to the total spectral power over the full 45-minute sequence, expressed as the ratio of powers (5-minute/45-minute). One dominant feature is the consistently lower ratio observed in the VLF band compared to either the LF or HF band. This difference is statistically significant in four of the nine segments (p<0.01). This lower value relative to the full 45-minute sample is likely mostly attributable to the shorter individual interval sampling time. Consequently, it suggests that five-minute sampling times are not ideal for characterizing overall VLF spectral intensity. Conversely, when VLF power is included in the analysis, the five-minute intervals tend to overestimate both LF and HF power contributions. On average, across all intervals, the ratios (mean±SD) for VLF, LF, and HF bands were 0.80±0.07, 1.20±0.11, and 1.22±0.10, respectively. The power ratios for the LF and HF bands are consistently greater, indicating values higher than expected for the full 45-minute sequence.

Estimation of MDC

Table 1 summarizes the SEME and the MDC determined for five-minute segment pairs separated by 10 minutes to simulate a hypothetical 10-minute pre-post intervention assessment of percentage change in spectral power. The average SEME across all six segment pairs ranged from 7.4±1.7% for the HF band to 10.7±1.9% for the VLF band. The corresponding MDC values ranged from 21.0±4.9% for the HF band to 30.4±5.5% for the VLF band. Tests for differences in MDC values among the frequency bands were not significant based on the nonparametric Friedman test (p=0.069). However, the decreasing value for the mean MDC trend from VLF to LF to HF bands is apparent. This suggests that, on average, detecting pre-post intervention changes requires a smaller change to exceed the MDC for the HF band compared to either the LF or VLF bands. Considering the MDC range among the segment pairs, the largest MDC was 37.1% for the VLF band, 27.2% for the LF band, and 27.5% for the HF band.

**Table 1:** Estimation of minimum detectible change in spectral power The minimal detectible change (MDC) is determined for five-minute segment pairs separated by 10 minutes to simulate a 10-minute pre-post intervention assessment of percentage change in spectral power. SEME is the standard error of the measurement, and MDC=2×√2×SEME. AVG is the average over all six segment pairs, and SD is the associated standard deviation. VLF, LF, and HF correspond to the very low, low, and high-frequency bands. SEME and MDC units are percentages.

Segments	VLF		LF		HF	
	SEME	MDC	SEME	MDC	SEME	MDC
1-4	9.3	26.4	9	25.5	9.4	26.6
2-5	12.5	35.2	9.6	27.2	6.8	19.1
3-6	10.3	29.2	9.4	26.6	7	19.8
4-7	8	22.6	8.9	25.1	5.8	16.5
5-8	13.1	37.1	9.4	26.5	9.7	27.5
6-9	11.3	31.9	8.2	23.2	5.8	16.4
AVG	10.7	30.4	9.1	25.7	7.4	21
SD	1.9	5.5	0.5	1.4	1.7	4.9

## Discussion

Variability in absolute spectral power among intervals

One aspect was to determine if there was a statistically significant difference among the intervals in either total spectral power or the distribution of that power within the frequency bands considered: VLF, LF, and HF. The findings indicated that neither total power nor power within any of the frequency bands statistically differed among intervals. This is a useful result since it provides additional confidence that similar spectral features would be expected for short-term samples drawn at different times during the assessment process. The lack of significant differences contributes to confidence in the use of five-minute intervals for short-term HRV assessments.

Maximum percentage changes among intervals

The present findings also offer an estimate of the maximum expected variations among the short-term interval powers, even though overall statistically significant variations were not found. For total spectral power, this “worst case” variation was 7.85%. For the separate frequency bands, it ranged from 3.75% for the VLF band to 8.5% for the LF band. This is a useful finding since it provides an initial estimate of the amount of change that could possibly be viewed as real subsequent to an intervention in which pre- and post-intervention HRV powers are used for assessment [24,25]. These low percentage differentials among intervals also contribute to confidence levels in the use of the five-minute intervals.

Differences between 5- and 45-minute intervals

The second major finding relates to differences between short-term spectral features and the full 45-minute interval. Significant differences were found between spectral power distributions, with VLF power within five-minute intervals being significantly less than for the full 45-minute sample, and LF and HF power being greater. As previously noted, the lower value obtained for the VLF band compared to the full 45-minute sample is likely due to the shorter sampling time (300 seconds). This would be a consequence of the inverse relationship between the sample duration and frequency content. For a 300-second sample, the LF cutoff (1/300) is 0.003 Hz, whereas for the 45-minute sample, it is 0.00037 Hz (1/2700). Thus, more VLF power would be expected to be included when a longer sample time is utilized. This finding suggests that five-minute sampling times are not ideal for characterizing overall VLF spectral intensity. The importance of this finding is that it reinforces the concept that when HRV frequency parameters are used to assess HRV status, care must be exercised in the sampling duration.

MDC aspects

The third finding relates to the estimation of the SEME and its associated MDC to be expected for a pre-post assessment of changes in the percentage of spectral power. This estimate was not based on an actual intervention but on a hypothetical intervention assumed to last 10 minutes. The purpose was to provide an initial estimate that could help in the planning of subsequent interventional research. When the overall average MDC was considered for each frequency band, it was found that the VLF band tended to have the largest MDC (31.4%), and the HF band tended to have the least (21%). The utility of these MDC values is that they provide an initial basis for estimating the percentage change in each of the spectral bands that might be interpreted as being a change attributable to an intervention, rather than only due to measurement or random error. However, as pointed out subsequently, the number of subjects from which these estimates are derived is small. 

Physiological processes impacting HRV parameters

The present focus on the spectral features of HRV analysis is prompted by their wide use in the clinical and research literature, in which the separate frequency bands (VLF, LF, and HF) are interpreted differently with respect to the physiological processes involved [26]. Spectral power is most often determined by Fourier or autoregressive methods [27]. Early work suggested that the LF band is largely reflective of baroreceptor, sympathetically mediated blood pressure control, whereas the HF band is mainly affected by respiration via changes in vagal afferent neural activity to the sinoatrial node, the natural cardiac pacemaker. Consequently, the concept of sympathetic-vagal information being represented by the LF/HF power ratio emerged [28,29]. Although an extensive discussion of the complexities and interactions that determine HRV is well beyond the scope of the present study, it has recently been reported that transient elevations in blood pressure, which trigger baroreceptor reflexes, are accompanied by increases in power in all three frequency bands [30]. The physiological basis of the VLF band is less clear. Some evidence has indicated it may be related to actions associated with the renin-angiotensin system [31]. This follows from the fact that the administration of an inhibitor of angiotensin-converting enzyme increased VLF power by about 20%, with no apparent effect on either LF or HF power. However, it was suggested that the main determinant of VLF power was parasympathetic related. Although changes in VLF power have been associated with a variety of conditions, the mechanisms remain unknown [32-35].

Parameter value comparisons

It is of interest to compare the present quantitative findings with the relevant data available in the literature. Adlakha et al. reported the total spectral power in a group of medical students at rest to be (mean±SD) 4900±4520 ms² [18]. The mean value reported therein is consistent with what was measured in the present group of medical students (4561±1434 ms²), who were about the same age, although the between-subjects SD was lower in the present group. Pinna et al. [36], using test-retest data obtained from a five-minute sample on two separate days in 39 healthy supine subjects, found no statistically significant difference in LF or HF power between consecutive days when paced breathing was used. They also estimated the number of subjects needed to detect a significant change in mean values of a spectral parameter, based on a criterion that to be clinically meaningful, the change should be ≥30% of the between-subjects SD. If that criterion were to be applied to the present total power data, a meaningful change of 430 ms² would be required. Žunkovič et al. [37] measured LF and HF power in 50 young healthy adults using five sequential five-minute samples, each separated by five minutes over 50 minutes of recording. These samples were not contiguous; the spectral powers were reported in logarithmic form, and subjects were seated, so a direct comparison is not possible. However, they found a nonsignificant effect of time (interval) on HF power (p=0.501), a result that is consistent with the present findings, but they also reported a significant effect for LF power (p=0.001).

Study limitations

The present findings apply specifically to the young adult, healthy population herein studied, and potential generalizations to either older populations or persons with cardiovascular-related conditions need verification. This follows due to reported differences in HRV parameters between cardiovascular-healthy and not-healthy groups [38] and also reported age-related differences [39]. However, the present findings provide baseline estimates of the likely type of variance in total and spectral power distributions among sequential short-term intervals during a single session. The findings also provide an initial estimate of the MDC in spectral power percentages.

Another limitation was the rather small number of subjects evaluated, which required the use of nonparametric statistics. It may be that if a much larger group were subsequently used, the use of parametric statistics with higher power for detecting differences were used an overall difference in power among intervals. Thus, a study with a larger number of participants is warranted to address this issue. However, until such results become available, the assumption is that the differences would not likely be large, since the worst-case maximums herein determined were at most 8.5% for all comparisons. A larger number of subjects might also result in somewhat different estimates of MDC, but until such results are reported, the present results provide initial estimates.

## Conclusions

The present findings provide initial estimates of the expected variations in HRV spectral power in the VLF, LF, and HF bands within contiguous five-minute segments, as well as an estimate of the minimum detectible “real” change between intervals separated by 10 minutes during a 45-minute session. These estimates include total spectral power and the percentage of power within each band, and they provide data regarding the pattern of variation. These initial estimates may also be useful in experimental planning and data interpretation, particularly when HRV spectral power changes are assessed following a short-duration intervention during a single session. The estimate of the MDC, which ranges from 21.0% for the HF band to 30.4% for the VLF band, provides useful information for estimating the number of participants needed to evaluate the impact of an intervention on the spectral components of HRV.

## References

[1] Gąsior JS, Hoffmann B, Silva LE, Małek Ł, Flatt AA, Baranowski R, Werner B (2020). Changes in short-term and ultra-short term heart rate, respiratory rate, and time-domain heart rate variability parameters during sympathetic nervous system activity stimulation in elite modern pentathlonists-a pilot study. Diagnostics (Basel).

[2] Eguchi K, Aoki R, Yoshida K, Yamada T (2017). Reliability evaluation of R-R interval measurement status for time domain heart rate variability analysis with wearable ECG devices. Annu Int Conf IEEE Eng Med Biol Soc.

[3] Kleiger RE, Stein PK, Bosner MS, Rottman JN (1992). Time domain measurements of heart rate variability. Cardiol Clin.

[4] Garbilis A, Mednieks J (2024). Differences in heart rate variability in the frequency domain between different groups of patients. Medicina (Kaunas).

[5] Pernice R, Faes L, Kotiuchyi I, Stivala S, Busacca A, Popov A, Kharytonov V (2019). Time, frequency and information domain analysis of short-term heart rate variability before and after focal and generalized seizures in epileptic children. Physiol Meas.

[6] Ori Z, Monir G, Weiss J, Sayhouni X, Singer DH (1992). Heart rate variability: frequency domain analysis. Cardiol Clin.

[7] Moghtadaei M, Dorey TW, Rose RA (2022). Evaluation of non-linear heart rate variability using multi-scale multi-fractal detrended fluctuation analysis in mice: Roles of the autonomic nervous system and sinoatrial node. Front Physiol.

[8] Dubey P, Verma M, John NA, Gutch M (2020). Nonlinear parameter of heart rate variability can diagnose early cardiac autonomic neuropathy. J Arrhythm.

[9] Voss A, Kurths J, Kleiner HJ, Witt A, Wessel N (1995). Improved analysis of heart rate variability by methods of nonlinear dynamics. J Electrocardiol.

[10] Brinth LS, Jørgensen T, Mehlsen J (2024). Normative values of short-term heart rate variability in a cross-sectional study of a Danish population. The DanFunD study. Scand J Public Health.

[11] Santos-de-Araújo AD, Bassi-Dibai D, Camargo PF (2023). Inter- and intrarater reliability of short-term measurement of heart rate variability on rest in chronic obstructive pulmonary disease (COPD). Heart Lung.

[12] Uhlig S, Meylan A, Rudolph U (2020). Reliability of short-term measurements of heart rate variability: findings from a longitudinal study. Biol Psychol.

[13] Burma JS, Graver S, Miutz LN, Macaulay A, Copeland PV, Smirl JD (2021). The validity and reliability of ultra-short-term heart rate variability parameters and the influence of physiological covariates. J Appl Physiol.

[14] Kim JW, Seok HS, Shin H (2021). Is ultra-short-term heart rate variability valid in non-static conditions?. Front Physiol.

[15] Shaffer F, Meehan ZM, Zerr CL (2020). A critical review of ultra-short-term heart rate variability norms research. Front Neurosci.

[16] Attreed A, Morand LR, Pond DC, Sturmberg JP (2024). The clinical role of heart rate variability assessment in cognitively impaired patients and its applicability in community care settings: a systematic review of the literature. Cureus.

[17] Bravi A, Longtin A, Seely AJ (2011). Review and classification of variability analysis techniques with clinical applications. Biomed Eng Online.

[18] Adlakha K, Mathur MK, Datta A, Kalsi R, Bhandari B (2023). Short-term effect of spiritual music on heart rate variability in medical students: a single-group experimental study. Cureus.

[19] Canino MC, Dunn-Lewis C, Proessl F (2022). Finding a rhythm: relating ultra-short-term heart rate variability measures in healthy young adults during rest, exercise, and recovery. Auton Neurosci.

[20] Lalitha S, Maheshkumar K, Shobana R, Deepika C (2020). Immediate effect of Kapalbhathi pranayama on short term heart rate variability (HRV) in healthy volunteers. J Complement Integr Med.

[21] Tarvainen MP, Niskanen JP, Lipponen JA, Ranta-Aho PO, Karjalainen PA (2014). Kubios HRV--heart rate variability analysis software. Comput Methods Programs Biomed.

[22] Atkinson G, Nevill AM (1998). Statistical methods for assessing measurement error (reliability) in variables relevant to sports medicine. Sports Med.

[23] Weir JP (2005). Quantifying test-retest reliability using the intraclass correlation coefficient and the SEM. J Strength Cond Res.

[24] Prinz-Zaiss M, Yeap AN, Moguilevski V, Trigg L, McGrath BP (1995). Power spectral analysis of heart rate variability during graded head-up tilting in patients with vasodepressor syncope. Clin Exp Pharmacol Physiol.

[25] Kamada T, Sato N, Miyake S, Kumashiro M, Monou H (1992). Power spectral analysis of heart rate variability in type As during solo and competitive mental arithmetic task. J Psychosom Res.

[26] Shaffer F, Ginsberg JP (2017). An overview of heart rate variability metrics and norms. Front Public Health.

[27] Badilini F, Maison-Blanche P, Coumel P (1998). Heart rate variability in passive tilt test: comparative evaluation of autoregressive and FFT spectral analyses. Pacing Clin Electrophysiol.

[28] Kamath MV, Fallen EL (1993). Power spectral analysis of heart rate variability: a noninvasive signature of cardiac autonomic function. Crit Rev Biomed Eng.

[29] Pagani M, Lombardi F, Guzzetti S (1984). Power spectral density of heart rate variability as an index of sympatho-vagal interaction in normal and hypertensive subjects. J Hypertens Suppl.

[30] Mitsuyama S, Sakamoto T, Nagasawa T, Kario K, Ozawa S (2024). A pilot study to assess the origin of the spectral power increases of heart rate variability associated with transient changes in the R-R interval. Physiol Rep.

[31] Taylor JA, Carr DL, Myers CW, Eckberg DL (1998). Mechanisms underlying very-low-frequency RR-interval oscillations in humans. Circulation.

[32] Ortiz-Guzmán JE, Mollà-Casanova S, Arias-Mutis ÓJ (2023). Differences in long-term heart rate variability between subjects with and without metabolic syndrome: a systematic review and meta-analysis. J Cardiovasc Dev Dis.

[33] Brusseau V, Tauveron I, Bagheri R (2022). Effect of hyperthyroidism treatments on heart rate variability: a systematic review and meta-analysis. Biomedicines.

[34] de Castilho FM, Ribeiro AL, Nobre V, Barros G, de Sousa MR (2018). Heart rate variability as predictor of mortality in sepsis: a systematic review. PLoS One.

[35] Shaffer F, McCraty R, Zerr CL (2014). A healthy heart is not a metronome: an integrative review of the heart's anatomy and heart rate variability. Front Psychol.

[36] Pinna GD, Maestri R, Torunski A, Danilowicz-Szymanowicz L, Szwoch M, La Rovere MT, Raczak G (2007). Heart rate variability measures: a fresh look at reliability. Clin Sci (Lond).

[37] Žunkovič B, Kejžar N, Bajrović FF (2023). Standard heart rate variability parameters-their within-session stability, reliability, and sample size required to detect the minimal clinically important effect. J Clin Med.

[38] Zhao Y, Yu H, Gong A, Zhang S, Xiao B (2024). Heart rate variability and cardiovascular diseases: a Mendelian randomization study. Eur J Clin Invest.

[39] Geovanini GR, Vasques ER, de Oliveira AR (2020). Age and sex differences in heart rate variability and vagal specific patterns - Baependi heart study. Glob Heart.

